# Closed-system manufacturing of therapeutic NK cells using automated cell enrichment and concentration processes enables scalable, robust and cost-effective solutions

**DOI:** 10.3389/fbioe.2025.1586912

**Published:** 2025-06-25

**Authors:** Nina Kok, Zoë Dekkers, Anica Loos, Thomas Bos, Puck van den Broek, Inge Post, Bertine Niessen, Jan Spanholtz, Volker Huppert, Monica Raimo

**Affiliations:** Glycostem Therapeutics, Oss, Netherlands

**Keywords:** NK cell therapy, process automation, cell therapy manufacturing, CliniMACS Prodigy, GMP, good manufacturing practice, closed system manufacturing, allogeneic cell therapy

## Abstract

**Background:**

Cell therapy is a promising field with the potential to improve outcomes for cancer and autoimmune disease patients. However, its complex and resource-intensive manufacturing requires costly infrastructure. Current processes, adapted from academic research, involve open handlings, manual processing, and repurposed equipment from biologics applications, raising quality and safety risks while increasing manufacturing costs. Closed, automated manufacturing systems can overcome these limitations.

**Methods:**

Allogeneic therapeutic natural killer (NK) cells can be generated from umbilical cord blood (UCB)-derived CD34^+^ hematopoietic stem cells in a closed, semi-automated process. Here, the automated CliniMACS Prodigy^®^ system has been evaluated for reliability and performance in two unit operations, the enrichment of CD34^+^ stem cells from cord blood and the final product harvest and concentration. In this study, we report the outcome of these two processes across N = 36 different manufacturing runs performed during process development and GMP manufacturing for clinical release.

**Results:**

Efficiency of CD34^+^ stem cell enrichment was evaluated across cord blood units with low (<4.50E06 CD34^+^ cells/unit, N = 11), medium (4.50-7.00E06 CD34^+^ cells, N = 13) or high (>7.00E06 CD34^+^ cells, N = 12) content. Robust performance was observed in all groups, with average CD34^+^ cell recoveries of 68.18%, 68.46%, and 71.94%, respectively. Higher purity was achieved in the high group (69.73%), compared to low (57.48%) and medium (62.11%). Factors such as UCB age, total nucleated cell count, and platelet or red blood cell content had no significant impact. For the final harvest and concentration process, we analyzed batches with low (<2 L, N = 7), medium (2–5 L, N = 14) or high cell culture volume (>5 L, N = 8). Approximatively 20% cell loss was reported, with average yields of 74.59% for low, 82.69% for medium, and 83.74% for high volumes. NK cell purity was stable at over 80%, and B and T cell impurities content remained low or undetectable.

**Discussion:**

This study presents an agile solution using a single piece of equipment during two steps of a complex NK cell manufacturing process. This approach ensures closed system safety, automation, high consistency, and cost-effectiveness, enabling the successful delivery of high-quality allogeneic NK cell therapies to patients.

## 1 Introduction

Over the last decades, cutting-edge developments in cell therapy manufacturing have led to a surge in the number of clinical trials and to the first marketing authorizations. Despite such progress, substantial challenges remain and must be overcome to assure robust manufacturing of safe, cost-effective, and efficacious therapies at scale (de Almeida Fuzeta et al., 2020). As the field matures by learning from successes and mistakes of early innovators, more product developers are striving to design good manufacturing practice (GMP)-compliant processes with a focus on scalability and commercialization from the earliest stages of process development. Equipment systems used in cell therapy manufacturing are often repurposed from other applications, such as bioreactors or apheresis devices (Lin-Gibson et al., 2017). Additionally, manufacturing still heavily relies on open handlings and manual processing and is therefore susceptible to microbiologic contamination and human error (Shah et al., 2023). Closed manufacturing systems minimize contamination risks, protect personnel from exposure to viral vectors and improve product consistency (Song et al., 2022; National Academies of Sciences Engineering and Medicine, 2024). The adoption of automated solutions improves efficiency, batch-to-batch reproducibility, minimizes operator error and reduces labor requirements (Park et al., 2024). When combined, these improvements enhance product quality and reduce manufacturing costs, thereby reducing failure rates by up to 75% (National Academies of Sciences Engineering and Medicine, 2024).

Scalable manufacturing technologies, consistent cell concentrations, fill volumes, and uniform quality from dose-to-dose are especially relevant for large-scale, allogeneic, “off-the-shelf” products (Lee et al., 2022; Park et al., 2024). Umbilical cord blood (UCB) is a valuable source for the development of cell therapies, as it contains highly proliferative hematopoietic stem and progenitor cells capable of multilineage differentiation (Park and Won, 2009). These qualities support the generation of large numbers of therapeutic cells of different types, including NK cells, and offer an advantage over sources like peripheral blood, which typically yield more mature and less proliferative cells (Domogala et al., 2017). Multiple NK cell and progenitor manufacturing methods have been described (Lamers-Kok et al., 2022), including from cord blood stem cells (Delaney et al., 2010; Spanholtz et al., 2011; Roeven et al., 2015). Glycostem Therapeutics’ first unmodified NK cell therapy has been evaluated earlier in a Phase I clinical trial as a fresh product (Dolstra et al., 2017) and more recently as part of a Phase I/II trial as a cryopreserved product (oNKord^®^, NCT04632316) in acute myeloid leukemia (AML) patients. Glycostem has developed a fully closed, semi-automated process for NK cell manufacturing within a class C clean room environment, called uNiK™. In this process, CD34^+^ hematopoietic stem cells (HSCs) are isolated from UCB, expanded and differentiated in large scale to NK cells, then harvested for cryopreservation of the final drug product. Within this process, we utilized a commercially available cell processing device, the Miltenyi CliniMACS Prodigy^®^ (Apel et al., 2013), to perform both the initial enrichment of CD34^+^ cells and the final harvest and concentration of NK cells. Thus, we 1) enabled automated cell processing in a closed system, 2) minimized reliance on multiple devices for unit operations, 3) improved process consistency and 4) facilitated its validation.

In this study, we reported for the first time the performance of the CliniMACS Prodigy^®^ in the enrichment of CD34^+^ cells from UCB, as it is designed for leukapheresis products (Apel et al., 2013), and in the concentration of the final NK cell product across over 30 manufacturing runs during process development activities and GMP manufacturing. We evaluated critical process parameters and product attributes associated with the enrichment and concentration processes, such as cell population composition and UCB age, or cell culture volume and impurity levels, respectively, that could influence process performance.

## 2 Materials and methods

### 2.1 Umbilical cord blood pre-process data collection

Fresh UCB units were supplied by Anthony Nolan Cord Blood (CB) Bank under ethical approval and with donor informed consent. Processing was performed at Glycostem Therapeutics – a recognized tissue establishment registered with the Dutch Health and Youth Care Inspectorate (Inspectie Gezondheidszorg and Jeugd, IGJ; registration number 16623 L/EW) – within 72 h after collection. UCB units were transported by temperature-controlled road freight (15°C–25°C) and were exempt from X-ray screening to preserve cell viability. This information was verified at receipt. Only UCB units containing ≥3.5E06 CD34^+^ cells for GMP batches and ≥2.0E06 CD34^+^ cells for R&D batches were considered eligible. Pre-process unit data, including weight, volume (although not provided for all units), total content of CD34^+^ cells, total content of nucleated cells (TNC), count of red blood cells (RBC)/µL and of platelets (PLT)/µL, were provided. Total RBC and PLT content were calculated by multiplying the volume by the cell concentration.

### 2.2 CD34^+^ hematopoietic stem cell enrichment

Enrichment of CD34^+^ HSCs from fresh UCB was performed using the LP-34 Enrichment Protocol (version 2.2, Miltenyi Biotech) on the CliniMACS Prodigy^®^. Through Prodigy Software guidance (version 1.4), the off-the-shelf single-use disposable designed for this process, i.e., the tubing set TS310 (Miltenyi Biotech), was installed. CliniMACS PBS/EDTA Buffer (Miltenyi Biotech) with 0.5% human serum albumin (HSA) (Sanquin) was used as washing buffer and proprietary Glycostem Basal Growth Medium (GBGM) (Glycostem Therapeutics) was used for cell elution. All enrichment protocols were performed according to “normal scale” specifications (up to 0.6E09 CD34^+^ cells and 60E09 total white blood cells), requiring one vial of CliniMACS CD34 reagent (Miltenyi Biotech). Fc receptor blocking was performed using a 5% IgG solution (Griffols Deutschland GmbH). From the eluted enriched fraction of approximatively 80 mL, a 1 mL sample was collected for quality control (QC) and flow cytometry analysis.

### 2.3 Cell culture

NK cell expansion and differentiation from the enriched CD34^+^ cells with the uNiK™ process were performed either under GMP conditions for clinical applications (N = 20, batches 1–22) or as part of R&D activities for process development purposes (N = 9, batches 25–36). The entire culture process lasted between 28–41 days. For GMP batches, a standardized cell culture protocol was followed, with early (day 0–12) cell expansion in static culture in gas-permeable bags (Vuelife 290AC, Saint-Gobain) in an incubator at 37°C and 5% CO_2_, followed by differentiation (day 13-end of culture) in continuous agitation in Xuri cellbags (2L or 10L basic cellbags, Cytiva) in a Xuri bioreactor at 37°C and 6% CO_2_. Cell culture days could vary by ± 1 day. The entire positive fraction obtained from CD34^+^ cell enrichment was seeded into one or two Vuelife bags. On day 13, cells were transferred to one or more Xuri bioreactors with a starting volume of 500 mL per bag. R&D batches were directly cultured in continuous agitation in Xuri cellbags (2L shot perfusion bags or 10L perfusion bags, Cytiva), starting with 300–500 mL per bag. Fresh medium was replenished twice a week. Throughout the entire culture process, cells were maintained in GBGM medium with 5%–10% human serum (Sanquin). During the expansion phase (day 0–12), cells were supplemented with FMS-like tyrosine kinase 3 ligand (Flt-3L), stem cell factor (SCF), interleukin-7 (IL-7) and thrombopoietin (TPO, replaced by IL-15 on day 8) (all from CellGenix). In the differentiation phase (day 13-end of culture), cells were supplied with IL-2 (Proleukin^®^, Clinigen Healthcare Ltd.), IL-7, IL-15 and SCF (CellGenix). A low-dose cytokine cocktail containing granulocyte macrophage colony-stimulating factor (GM-CSF, CellGenix), granulocyte CSF (G-CSF, Neupogen^®^, Amgen) and IL-6 (CellGenix) was supplemented through the whole culture (Spanholtz et al., 2011; Lamers-Kok et al., 2022).

### 2.4 Harvest and cell concentration

On harvest day, a pre-harvest 3 mL sample was collected from the cell culture for QC and flow cytometry analysis. Then, the cell suspension was washed and concentrated using the Cell Concentration Protocol (version 1.1, Miltenyi Biotech) on the CliniMACS Prodigy^®^. Through Prodigy software guidance (version 1.4), the off-the-shelf single-use disposable designed for this process, i.e., the tubing set TS720 (Miltenyi Biotech), was installed. A solution of 0.9% saline (Baxter BV) and 0.5% HSA (Sanquin) was used as washing buffer. The entire cell suspension was taken up in a single stage at a flow rate of 60 mL/min and washed over seven cycles at 2,500 rpm. Using standard reduction and a second chamber wash as parameters, the cell concentration process yielded 135 mL of concentrated cells in saline 0.9% with 0.5% HSA. The volume was then adjusted to 400 mL, from which a post-harvest 1 mL sample was collected for QC and flow cytometry analysis. To ensure product safety, samples were collected during harvest, concentration and final formulation for contaminant testing, i.e., sterility (according to Ph. Eur. 2.6.1), *mycoplasma* (according to Ph. Eur. 2.6.7) and endotoxin (according to Ph. Eur. 2.6.14). All batches met specification limits (data not shown).

### 2.5 Cell counting and flow cytometry

#### 2.5.1 Sample handling and flow cytometry

Samples collected after CD34^+^ cell enrichment or before (pre-harvest) and after (post-harvest) cell concentration were analyzed using flow cytometry on a CytoFLEX S or CytoFLEX LX device (Beckman Coulter). Sample acquisition and analysis were performed according to qualified QC standard operating procedures.

#### 2.5.2 CD34^+^ HSC enumeration

For all GMP batches and R&D batches 23–32, enumeration of CD34^+^ HSCs was performed using the *in vitro* diagnostics (IVD) CD34 Enumeration Kit (Miltenyi Biotech). Following the manufacturer’s recommendations, a 100 µL sample from the enriched fraction was stained with antibodies against CD34 (APC, clone AC136), CD45 (FITC, clone 5B1) and the 7-amino-actinomycin D (7-AAD) viability dye and incubated for 10 min at 4°C in the dark, then samples were measured using flow cytometry. For R&D batches 33–36, CD34^+^ cells were counted by staining 50 µL of cell suspension with 7-AAD and with antibodies against CD34 and CD45 (Table 1) for 15 min at room temperature (RT) in the dark. The final volume was filled to 100 µL with phosphate buffered saline (PBS), of which 50 µL was measured using flow cytometry.

**TABLE 1:** Overview of antibodies/stains used in flow cytometry methods. All antibodies were used according to supplier recommendations.

Antibody	Fluorochrome	Clone	Cat. No	Supplier	Used in batches
CD34	PC7	581	A21691	Beckman Coulter	R&D 33-36
CD45	Krome Orange	J33	B36294	Beckman Coulter	GMP 1-22, R&D 23-32
CD45	Brilliant Violet 510	2D1	368536	BioLegend	R&D 33-36
CD56	APC-A750	N901	B46024	Beckman Coulter	GMP 1-22, R&D 25-32
CD56	APC/Fire750	QA17A16	392408	BioLegend	R&D 33-36
CD3	Vioblue	BW264/56	170-081-046	Miltenyi	GMP 1-22, R&D 25-36*
CD19	PE	LT19	170-081-057	Miltenyi	GMP 1-22, R&D 25-36
7AAD	-	-	A07704	Beckman Coulter	GMP 1-22
7AAD	-	-	A9400-1MG	Sigma Aldrich	R&D 23-36

*CD3 was not used for cell count of R&D batches.

#### 2.5.3 Cell count, viability and purity analysis

For total and NK cell counts, 50 µL of cell suspension was stained with 7-AAD viability dye and with antibodies against CD45, CD56 and CD3 (Table 1) for 15 min at RT in the dark. The final volume was filled to 200 µL with PBS, of which 50 µL was measured using flow cytometry. For B and T cell impurity analysis, 400,000 cells were stained with 7-AAD, CD45, CD56, CD19 and CD3 (Table 1) for 15 min at RT in the dark. The final volume was filled to 200 μL with PBS before flow cytometry acquisition. The limit of quantification (LoQ) for impurity analysis was determined during assay qualification by spiking samples with a known number of B or T cells and ensures detection of impurities as low as 0.1% B or T cells per 100,000 acquired cells. Lower values were reported as “below LoQ.” A reference sample was included in all measurements to ensure adequate testing conditions.

### 2.6 Data analysis

#### 2.6.1 Software

CytExpert (Beckman Coulter, version 2.3.0.84) was used for data acquisition, visualization, and analysis on the CytoFLEX S or CytoFLEX LX flow cytometers. Additionally, Kaluza Analysis (Beckman Coulter, version 2.1.00003.20057) was used to visualize and analyse acquired data.

#### 2.6.2 Statistics

GraphPad Prism (version 10.4.0) was used for data visualization and statistical analysis. The specific test used in each analysis is indicated in the corresponding figure legend, with differences considered significant for p-values ≤0.05. For linear regression analysis, the line equation is reported, and the goodness of fit was assessed using the coefficient of determination *R* ^2^.

## 3 Results

### 3.1 The CliniMACS Prodigy^®^ enables robust automated closed system enrichment of CD34^+^ HSCs from umbilical cord blood, irrespectively of stem and other cells’ content or cord blood age

**FIGURE 1:**
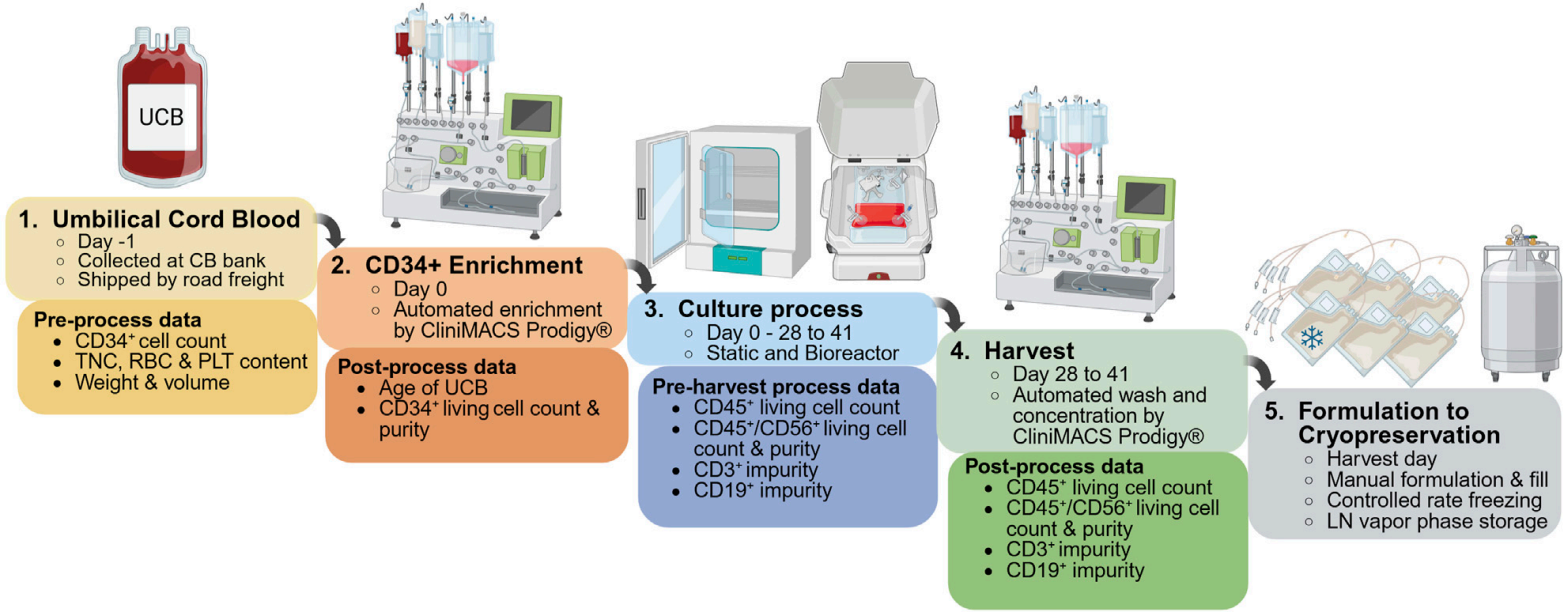
Glycostem Therapeutics’ uNiK™ platform for the manufacturing of NK cell therapies. This platform enables the production of clinical-grade, off-the-shelf NK cells within a fully closed, semi-automated system in a class C clean room environment. First, umbilical cord blood units are received from the cord blood bank. Next, CD34^+^ hematopoietic stem cells are enriched using the CliniMACS Prodigy^®^. These cells undergo expansion and differentiation culture for a period of 4–6 weeks (ranging between 28 and 41 days), initially in static or agitated conditions to promote NK progenitor expansion, followed by continuous agitation until full NK differentiation. At the end of the culture process (day 28–41), cells are harvested and concentrated using the CliniMACS Prodigy^®^. The final drug substance is then filled in cryobags, frozen under controlled conditions, then stored until shipped to treatment centers. Pre-process and post-process data analyzed in this study from the CD34^+^ cell enrichment and final product concentration processes are indicated. UCB: umbilical cord blood; TNC: total nucleated cells; RBC: red blood cells; PLT: platelets.

Glycostem’s closed system manufacturing process for the generation of therapeutic NK cells, uNiK™, comprises multiple steps, including two unit operations performed on the CliniMACS Prodigy®, i.e., enrichment of CD34^+^ HSCs from umbilical cord blood and final product harvest and concentration (Figure 1). Briefly, UCB units are collected and shipped from the cord blood bank, then processed at Glycostem within 72 h. After enrichment, CD34^+^ cells are cultured in a static system or in a rocking bioreactor in continuous agitation for the first 2 weeks (Supplementary Figure S1A), to promote the expansion of NK progenitors. For the NK differentiation phase, cells are transferred to or maintained in a rocking bioreactor, where they continue to expand and differentiate for 2–4 additional weeks (Supplementary Figure S1B). At the end of the cell culture process, NK cells are harvested and concentrated before the final product is filled into cryogenic bags and cryopreserved.

**FIGURE 2:**
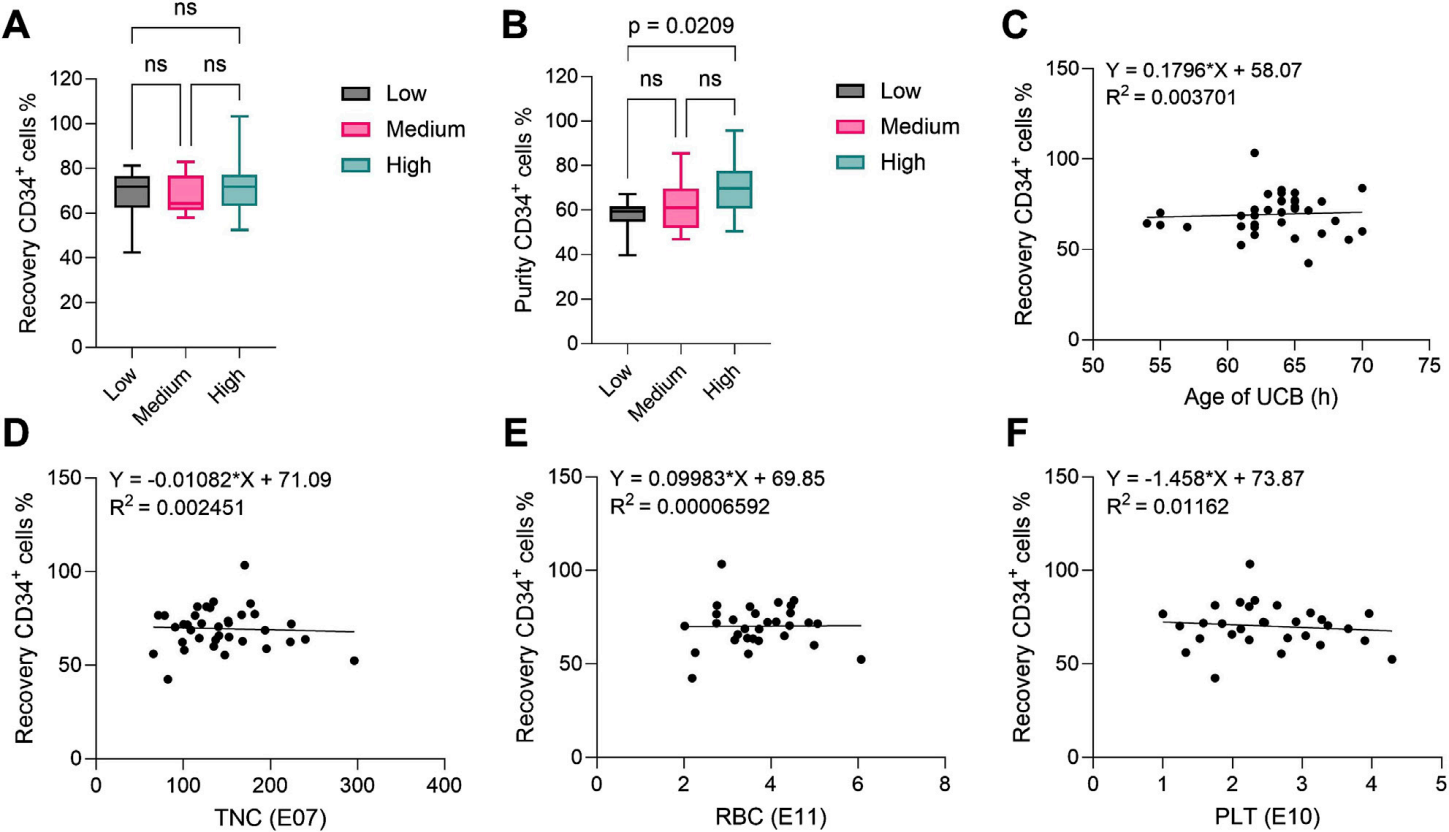
CD34^+^ hematopoietic stem cell recovery and purity after CD34^+^ cell enrichment process using the CliniMACS Prodigy^®^. In all plots, CD34^+^ cells were identified as CD34^+^CD45^dim^ living cells. **(A)** Recovery and **(B)** purity of CD34^+^ cells after cell enrichment from UCB units with low (n = 11), medium (n = 13) and high (n = 12) CD34^+^ content (reported by the supplier). The central line denotes the median; boxes delimitate the 25th and 75th percentiles and whiskers mark the minimum and maximum. Significance was determined using one-way ANOVA. **(C–F)** Linear regression analysis between **(C)** age of UCB (n = 35), **(D)** TNC (n = 36), **(E)** RBC (n = 30) and **(F)** PLT (n = 30) content in UCB as reported by the supplier and recovery of CD34^+^ hematopoietic stem cells after cell enrichment. All *R* ^2^ values show no correlation. UCB: umbilical cord blood; TNC: total nucleated cells; RBC: red blood cells; PLT: platelets.

The content of CD34^+^ HSCs in each UCB unit is determined by the cord blood bank before shipment and varies between units. To assess the performance of the CD34^+^ cell enrichment process with the Prodigy, we compared data from units with low (<4.50E06 CD34^+^ cells/unit, N = 11), medium (4.50-7.00E06 CD34^+^ cells, N = 13) and high (>7.00E06 CD34^+^ cells, N = 12) content of HSCs, with an average cord blood volume of 119.1 ± 19.0 mL (mean ± standard deviation (SD)). A summary of the relevant parameters assessed for all units is presented in Table 2. After Prodigy enrichment, we evaluated both the recovery and purity of CD34^+^ cells from low, medium and high-content units. Enumeration of CD34^+^ cells after enrichment was performed by flow cytometry and analyzed according to the guidelines established by the International Society of Hematotherapy and Graft Engineering (ISHAGE) (Sutherland et al., 1996) to identify living CD34^+^CD45^dim^ stem cells (gating strategy in Supplementary Figure S2). Recovery was calculated with the number of CD34^+^ cells retrieved after enrichment compared to initial content provided by the cord blood bank, while purity was determined as the proportion of CD34^+^ cells within the enriched population. We observed comparable recovery (Figure 2A) for all groups, with mean values of 68.18% ± 11.39%, 68.46% ± 8.60% and 71.94% ± 13.40% (mean ± SD), respectively. Although similar in all groups, purity was significantly higher in the high group (69.73% ± 11.80%) when compared to the low group (57.48% ± 7.55%), but not to the medium group (62.11% ± 10.98%) (Figure 2B). This data demonstrates that the CD34^+^ cell content in the cord blood does not impact the efficiency of the enrichment process. Next, we evaluated the impact of other parameters. Using linear regression analysis, we correlated the CD34^+^ cell recovery with the age of UCB at the time of isolation (within 72 h) (Figure 2C), with the total nucleated cell content (Figure 2D), with the red blood cell content (Figure 2E) and with the platelet content (Figure 2F), but we did not observe any correlation between these variables and process outcome. Similar results were observed when those parameters were correlated with the CD34^+^ cell purity (Supplementary Figure S3). Overall, the Prodigy CD34^+^ cell enrichment program demonstrated consistent robustness, irrespectively from the CD34^+^ cell content, from other cell types or from cord blood age (in the range evaluated), however a moderately higher purity of recovered CD34^+^ cells can be obtained from units containing a large (over 7E06) CD34^+^ cell population.

**TABLE 2:** Summary of the relevant parameters assessed for the CD34^+^ cell enrichment process using CliniMACS Prodigy^®^ for GMP or R&D purposes. Pre-process data provided by the Cord Blood (CB) bank consists of total content of CD34^+^ cells, total content of TNC, count of RBC/µL and PLT/µL, weight (in g) and volume (in mL) of the UCB. If volume was not provided, N/A is noted. For batch 25, delivery time exceeded the 72-h window; therefore, it was excluded from age correlation analysis and omitted from the mean calculation. Post-process data generated by Glycostem Therapeutics (GS) consists of age of UCB at the start of the enrichment process (in hours), CD34^+^ cell number, recovery and purity after enrichment.

Batch nr.		Pre-process data (CB Bank)						Post-process data (GS)			
		CD34^+^ cells (E06)	TNC (E07)	RBC (E06/µL)	PLT (E03/µL)	Volume (mL)	Weight (g)	Age of UCB (h)	CD34^+^ cells (E06)	Recovery (%)	CD34^+^ purity (%)
GMP batches	1	5.13	101.2	3.29	136	N/A	213	62	3.0	58.1	68.7
	2	4.57	78.5	2.55	114	N/A	111	65	3.5	76.6	48.6
	3	5.18	90.8	2.75	169	73.4	111	55	3.6	70.3	61.1
	4	4.41	99.1	2.37	158	N/A	78	62	2.8	62.4	58.6
	5	4.98	135.1	3.61	236	138.3	137	70	3.0	60.0	46.9
	6	12.33	296.5	3.82	270	159.0	159	61	6.5	52.5	70.6
	7	4.52	118.32	2.88	173	N/A	110	54	2.9	64.4	52.6
	8	12.26	141	3.57	219	90.7	95	68	8.1	65.8	74.0
	9	9.56	151.52	2.67	280	117.3	157	65	7.0	73.6	78.8
	10	5.97	195.63	3.31	198	N/A	177	67	3.5	58.8	70.7
	11	3.53	65.71	2.39	141	94.5	133	65	2.0	56.1	63.4
	12	7.1	134.9	3.94	202	115.0	155	70	6.0	83.9	50.4
	13	8.25	223	2.86	300	130.0	162	57	5.2	62.4	71.6
	14	4.79	140.42	3.35	255	132.3	163	64	3.4	70.6	65.8
	15	7.31	113.6	2.82	190	N/A	131	67	5.6	76.6	66.0
	16	8.37	182.04	3.25	228	137.0	178	65	6.5	77.3	61.9
	17	7.50	224.1	3.72	188	131.0	180	62	5.4	72.0	79.6
	18	7.92	194.08	3.26	185	114.4	154	61	5.4	68.7	69.2
	19	4.38	151.91	2.69	190	153.3	195	65	3.2	72.6	56.3
	20	6.44	137.34	3.32	142	108.0	148	55	4.1	63.5	70.7
	21	4.33	82.4	2.47	197	88.8	127	66	1.8	42.5	59.5
	22	4.95	167.13	2.77	301	131.5	172	64	3.8	77.0	60.9
R&D batches	23	5.76	116.35	2.75	175	100.2	139	64	4.7	81.3	85.6
	24	8.57	170.46	2.76	216	104.0	143	62	8.9	103.4	60.2
	25	4.11	121.19	3.24	202	121.0	161	>72	3.0	72.3	39.7
	26	3.75	152.6	3.23	229	133.4	174	64	2.4	65.1	61.7
	27	3.13	71.3	2.51	91	109.7	149	64	2.4	76.7	60.6
	28	7.82	105	3.65	133	139.0	180	66	5.6	71.6	95.7
	29	4.82	177.24	3.77	191	110.6	150	64	4.0	83.0	51.6
	30	2.56	108.82	3.02	325	112.5	152	62	1.8	68.8	49.1
	31	4.62	168.1	2.77	196	114.4	154	61	2.9	62.8	56.1
	32	3.72	130.76	3.00	191	117.3	157	63	3.0	80.6	67.1
	33	10.1	147.78	2.68	208	130.0	171	69	5.6	55.4	58.8
	34	4.43	126.69	3.57	211	125.0	166	65	3.6	81.3	54.8
	35	3.62	100.37	2.55	146	108.0	148	63	2.6	71.8	61.4
	36	6.27	240.25	2.62	211	132.0	173	62	4.0	63.8	68.3
Mean ± SD		**6.03 ± 2.43**	**143.36 ± 50.52**	**3.05 ± 0.45**	**200 ± 52**	**119.1 ± 19.0**	**152 ± 28**	**63 ± 4**	**4.2 ± 1.8**	**69.5 ± 11.0**	**63.2 ± 11.3**

TNC, total nucleated cells; RBC, red blood cells; PLT, platelets; UCB, umbilical cord blood; SD, standard deviation. Values in the last row represent the mean and standard deviation (SD).

### 3.2 The CliniMACS Prodigy^®^ is suitable for automated closed system concentration of CD56^+^ NK cells from a range of cell culture volumes, without affecting the composition or purity of the product

**FIGURE 3:**
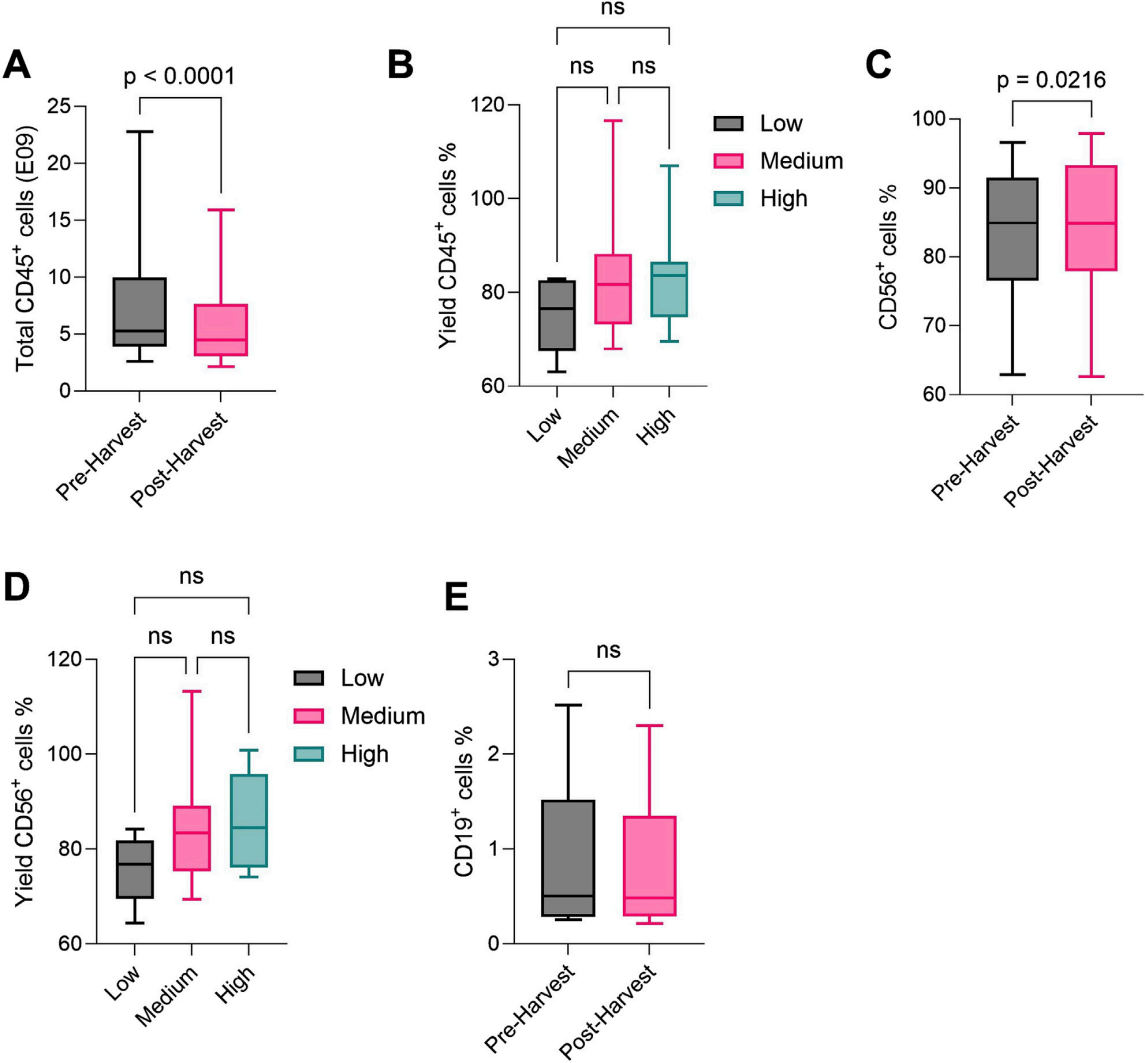
NK drug substance composition before and after the concentration process using the CliniMACS Prodigy^®^. **(A)** Total CD45^+^ cell (identified as CD45^+^ living) number before and after the cell concentration process (n = 29). **(B)** Total CD45^+^ cell yield after the cell concentration process, according to low (n = 7), medium (n = 14) or high (n = 8) cell culture volume. **(C)** CD56^+^ NK cell (identified as CD45^+^CD56^+^CD3^−^ living) purity before and after cell concentration process (n = 29). **(D)** CD56^+^ cell purity after cell concentration process, according to low (n = 7), medium (n = 14) or high (n = 8) cell culture volume. **(E)** CD19^+^ B cell impurity content (identified as CD45^+^CD19^+^ living) before and after cell concentration process (n = 7). For all plots, the central line denotes the median; boxes delimitate the 25th and 75th percentiles and whiskers mark the minimum and maximum. For statistical analysis, paired T-test was used in **(A, C, E)**, one-way ANOVA in **(B,D)**.

At the end of the culture process, differentiated NK cells (Supplementary Figure S1B) were harvested from bioreactor bags to be washed and concentrated with the CliniMACS Prodigy^®^ (Figure 1). We assessed the impact of the concentration process by evaluating the yield, defined as the percentage of total cells retrieved after concentration compared to pre-harvest levels. Additionally, we assessed NK purity and the levels of B and T cell impurities in the final product. To gain more insight on the effect of total cell numbers and culture volume on the concentration process, we included batches with volumes ranging from 800 mL to 12,800 mL, divided in low (<2 L, N = 7), medium (2–5 L, N = 14) or high volume (>5 L, N = 8). A summary of the concentration data is presented in Table 3. Cell yield, expressed as total CD45^+^ living cells (gating strategy in Supplementary Figure S4), was 5.74E09 ± 3.58E09 (mean ± SD), significantly lower than the 7.19E09 ± 4.75E09 cells counted before harvest (Figure 3A). When comparing cell yield for the different culture volumes, we observed similar results, however with a slightly lower efficiency for low culture volumes (74.59% ± 7.63%), compared to medium (82.69% ± 12.58%) or high (83.74% ± 11.35%) (Figure 3B). NK cell purity, determined as the ratio of CD45^+^CD56^+^CD3^−^ living cells (Supplementary Figure S4), slightly increased from 83.07% ± 9.25% before harvest to 84.53% ± 9.96% after harvest (Figure 3C). Furthermore, higher cell culture volumes were associated with a slight increase in NK cell content (85.83% ± 9.90% in high), compared to low (75.69% ± 7.33%) or medium (83.54% ± 11.45%) volumes (Figure 3D). During product release testing, T and B cell impurities were evaluated before and after the concentration process. While T cell content (defined as CD45^+^CD3^+^ living cells) was below the assay’s LoQ for all batches (data not shown), B cells (defined as CD45^+^CD19^+^ living cells, Supplementary Figure S5) were detected for some batches (N = 7). However, the B cell content was very low and remained unchanged before (0.88% ± 0.85%) and after (0.83% ± 0.75%) harvest (Figure 3E). Taken together, these results demonstrate that, despite a ∼20% cell loss, the Prodigy concentration process shows robust performance across different culture volumes without compromising the composition or purity of NK cell products.

**TABLE 3:** Summary of relevant parameters assessed for the NK cell concentration process using CliniMACS Prodigy^®^ for GMP or R&D purposes. Total CD45^+^ cell number, CD56^+^ (%) and CD19^+^ (%) content is reported before (pre-harvest) and after (post-harvest) the cell concentration process. Cell culture volume (in mL) was measured pre-harvest. CD19 content below the established limit of quantification is mentioned as “below LoQ”. LoQ: limit of quantification. Independent R&D batches generated from the same donor but cultured and harvested separately are distinguished with letters.

Batch nr.		Pre-harvest				Post-harvest			
		Total CD45^+^ cells (E09)	Volume (mL)	CD56^+^ (%)	CD19+ (%)	Total CD45^+^ cells (E09)	Yield CD45^+^ cells (%)	CD56^+^ (%)	CD19^+^ (%)
GMP batches	1	11.00	9990	90.07	below LoQ	9.00	72.6	91.86	below LoQ
	2	15.90	9120	81.65	below LoQ	13.70	86.3	85.05	below LoQ
	3	12.90	8930	74.87	below LoQ	11.10	86.2	84.90	below LoQ
	4	3.38	3690	63.23	0.285	2.96	87.6	63.69	0.210
	5	2.93	3380	75.55	below LoQ	2.16	73.6	71.54	below LoQ
	7	2.61	2770	91.76	below LoQ	2.33	89.4	94.41	below LoQ
	8	4.61	3460	86.35	below LoQ	5.38	116.6	83.84	below LoQ
	9	11.30	6660	70.54	below LoQ	9.78	86.6	70.37	below LoQ
	10	4.99	5580	78.56	below LoQ	5.34	106.9	74.06	below LoQ
	11	3.84	2400	84.91	1.520	3.60	93.8	82.87	1.350
	12	8.34	4690	72.07	below LoQ	6.91	82.8	73.49	below LoQ
	13	5.31	4030	77.56	below LoQ	4.66	87.8	74.11	below LoQ
	14	5.38	1690	85.38	0.300	4.46	82.9	86.74	0.400
	15	11.50	5140	80.88	below LoQ	9.31	80.9	82.55	below LoQ
	16	6.79	5240	62.92	below LoQ	5.48	80.7	62.61	below LoQ
	17	5.56	2900	77.86	below LoQ	4.00	71.9	83.25	below LoQ
	18	22.80	12850	89.15	0.250	15.90	69.5	95.77	0.290
	19	8.35	4000	89.35	2.515	5.68	68.0	91.25	2.300
	20	5.17	4400	78.39	0.820	4.31	83.4	82.39	0.810
	22	3.96	2930	92.63	0.500	3.04	76.8	93.30	0.480
R&D batches	25a	5.25	910	81.47	below LoQ	4.12	78.6	84.88	below LoQ
	25b	3.46	840	93.62	below LoQ	2.18	63.0	95.75	below LoQ
	30	14.50	4950	91.26	below LoQ	10.00	69.0	93.33	below LoQ
	34a	3.06	4960	75.27	below LoQ	2.33	76.2	81.79	below LoQ
	34b	8.99	4960	96.58	below LoQ	7.24	80.5	97.87	below LoQ
	36a	4.32	870	94.98	below LoQ	3.07	71.1	95.90	below LoQ
	36b	4.15	959	93.77	below LoQ	2.80	67.5	96.63	below LoQ
	36c	3.82	959	85.79	below LoQ	3.16	82.6	84.45	below LoQ
	36d	4.48	958	92.54	below LoQ	3.43	76.6	92.79	below LoQ
Mean ± SD		**7.19 ± 4.75**	**4283 ± 2975**	**83.07 ± 9.25**	**0.884 ± 0.848**	**5.77 ± 3.60**	**81.0 ± 11.5**	**84.53 ± 9.96**	**0.834 ± 0.754**

SD: standard deviation. Values in the last row represent the mean and standard deviation (SD).

### 4 Discussion

Allogeneic NK cell therapies represent a highly promising approach for the treatment of hematological malignancies, solid tumors, and autoimmune diseases. NK cells have demonstrated excellent safety profiles and encouraging efficacy in Phase I/II oncology trials, both as non-engineered products and engineered CAR-NK cells, including in combination with monoclonal antibodies or engagers (Lamers-Kok et al., 2022; Shi et al., 2024). Emerging clinical applications in autoimmune diseases–such as systemic lupus erythematosus, lupus nephritis and myasthenia gravis–further support the expansion of NK cells’ therapeutic scope (Kong et al., 2025). The development of automated, scalable NK manufacturing platforms allows for the production of hundreds of doses per batch, enabling broader global access to cell therapies at sustainable costs and overcoming key limitations associated with current autologous CAR-T approaches.

Currently, therapeutic demand for commercial cell therapies exceeds supply (National Academies of Sciences Engineering and Medicine, 2024). As of early 2024, 34,000 patients have received commercially available CAR-T immunotherapies worldwide (Reville, 2024), but about 350,000 patients are projected to be treated with 30–60 cell and gene therapy products by 2030 (Quinn et al., 2019). Automated closed manufacturing systems provide a solution for increasing patient access to cell therapies by minimizing opportunities for contamination and operator error, thereby reducing failure rate, improving product quality, and lowering manufacturing costs (National Academies of Sciences Engineering and Medicine, 2024). Early implementation of closed system solutions from process development stages facilitates regulatory adherence. The U.S. Food and Drug Administration (FDA) encourages the adoption of closed manufacturing systems to establish adequate facility and equipment standards and monitoring plans (Wonnacott, 2023). High standardization can also accelerate investigational new drug (IND) application filing and reduce regulatory review efforts for the chemistry, manufacturing and controls (CMC) package (National Academies of Sciences Engineering and Medicine, 2024). Despite their advantages, automated closed solutions involve a trade-off between long-term operational cost reduction and high upfront capital expenditures. While long-term benefits outweigh the initial investments, these costs often pose significant barriers for small biotech companies and academic laboratories (Ten Ham et al., 2021).

Glycostem Therapeutics has developed an innovative process for closed and semi-automated manufacturing of allogeneic NK cell therapies at scale. To enable automated processing and optimize cost-efficiency, we consolidated two critical manufacturing steps, the isolation of CD34^+^ stem cells from cord blood and the final product harvest and concentration, into a single platform using the Miltenyi CliniMACS Prodigy^®^. This is the first report of CD34^+^ hematopoietic stem cell enrichment from umbilical cord blood using Prodigy, as it is designed for enrichment from mobilized leukapheresis products, e.g., in the context of HSC transplantation (Stroncek et al., 2016). Nevertheless, the process demonstrated robust performance, with consistent cell yield and purity across multiple units. Acquisition of UCB units from the cord blood bank is solely based on their content of CD34^+^ cells and feasibility of delivery within 72 h from birth. Parameters such as CD34^+^ cell content, total nucleated cells, red blood cells, and platelets vary between units. However, our study showed that the enrichment process output was not affected by such variations, maintaining consistent performance across many units. Additionally, UCB age did not impact the quality of the process or of the cell population. This is in line with previous reports, where despite UCB storage time affected leukocyte viability, CD34^+^ cell content remained stable (Salge-Bartels et al., 2009). Recovery of the CD34^+^ population averaged approximatively 70% and was not affected by low, medium or high content levels, while purity, ranging on average between 57% and 70%, positively correlated with higher stem cell content. The modest decrease in purity observed from UCB units with lower CD34^+^ cell content may result from reduced efficiency of labelling and magnetic separation, which can occur when a higher proportion of non-target cells is present. Additionally, a fraction of CD34^+^ cells might remain trapped in the magnetic column and not be fully eluted–an event that could disproportionately affect low-content units. Importantly, this variability has minimal impact on our manufacturing process, as we apply a predefined threshold of 3.5E06 CD34^+^ cells per cord blood to ensure that only units meeting minimum input criteria are processed. Other studies, evaluating CD34^+^ cell recovery from peripheral blood, obtained average recovery rates of 51%–74% using Prodigy and of 44%–77% using the semiautomated CliniMACS Plus^®^ instrument (Keever-Taylor et al., 2012; Dvorak et al., 2013; Spohn et al., 2015; Stroncek et al., 2016). Only one study from Pello et al., reported 100% CD34^+^ recovery from pooled donor products, though observations were limited to a single execution (Pello et al., 2020). Our process and study are currently limited to UCB processing within 54–70 h after birth; shorter time intervals might further increase yield and quality. Additionally, the comparability of data obtained from different laboratories is an important consideration for the interpretation of the outcome of the enrichment process. In this study, CD34^+^ cell recovery was evaluated using mixed data sets, with pre-process CD34^+^ counts measured at the cord blood bank and post-process yields measured in our QC laboratory. Although both facilities employed flow cytometry-based quantification following ISHAGE guidelines (Sutherland et al., 1996), interlaboratory variability – including differences between instrumentation, gating strategies, and operators – may influence the comparability of the results (Ivana Ferrero et al., 2023). Such methodological differences can impact data interpretation and should be carefully considered when analyzing recovery outcomes.

Final cell product harvest and concentration similarly exhibited high robustness across different cell culture volumes, indicating that differences in harvest duration–influenced by the processed volume–did not compromise cell recovery. Total cell recovery averaged approximatively 80%, with some cell loss likely attributable to removal of dead or dying cells, which can contribute to an increased post-harvest viability. Notably, our recovery rates remain substantially higher than those previously reported, with 47%–62% recovery of regulatory T cells obtained using the Prodigy system and 66% using the Miltenyi CliniMACS^®^ Plus, when culturing cells at densities up to 8.5E06 cells/mL (Marin Morales et al., 2019), or 51% recovery of T cells reported with the Prodigy (Zhu et al., 2016). Higher cell densities can negatively impact recovery due to accumulation of debris, proteins and free DNA; therefore, optimization of the harvest cell density is recommended to minimize loss. Other contributing factors include multiple centrifugation steps, centrifugation imbalances, and variations in handlings – which particularly affect open, manual processes that are more prone to operator variability. Furthermore, reducing flow rates during processing and utilizing pre-coated, low-adhesion collection bags could reduce shear stress and minimize cell adhesion to surfaces, potentially improving overall recovery. Importantly, CD56^+^ NK content and product purity, assessed via QC testing before and after harvest, remained stable at over 80%, though we observed a slight trend toward higher yield with larger cell culture volumes. B and T cell impurities remained low or undetectable. Potency testing of the drug substance post-harvest demonstrated robust cytotoxic activity of the NK cell products, with over 90% average lysis of K562 target cells at a 10:1 effector-to-target ratio (data not shown), confirming the effectiveness of the process in generating functional, high-quality NK cells. Additionally, closed system manufacturing ensured consistent absence of contamination in the final product, as all GMP batches passed sterility, *mycoplasma*, and endotoxin testing (data not shown), and were deemed safe for clinical use.

Despite the robustness of the current workflow, opportunities for process optimization remain, particularly in reducing manual operations and streamlining documentation. Transitioning from paper-based to electronic batch records (EBRs) could improve efficiency of batch review and deviation management. Additionally, automating medium exchange and fill-and-finish would minimize hands-on time and support scalability for clinical and commercial applications.

### 5 Conclusion

The growing demand for commercial cell therapies manufactured at scale necessitates efficient and cost-effective manufacturing processes. Early adoption of automated closed systems provides long-term benefits regarding safety, quality, cost reduction, and regulatory compliance. Innovative, versatile approaches can help offset initial investment costs while improving operational efficiency. Glycostem Therapeutics has successfully implemented this strategy by leveraging one device, the Miltenyi CliniMACS Prodigy^®^, for both CD34^+^ enrichment from UCB and final cell product harvest and concentration. High consistency, reduced workload, and lower facility costs support broader patient access to innovative and necessary treatments.

## Data Availability

The original contributions presented in the study are included in the article/Supplementary Material, further inquiries can be directed to the corresponding author.
