# Supplementary material for: Closed-system manufacturing of therapeutic NK cells using automated cell enrichment and concentration processes enables scalable, robust and cost-effective solutions
Source: Front Bioeng Biotechnol. 2025 Jun 25;13:1586912. doi: 10.3389/fbioe.2025.1586912 (PMC12238007; doi:10.3389/fbioe.2025.1586912)
Supplement: Supplementary file 1 [file Supplementaryfile1.pdf]

Closed-system manufacturing of therapeutic NK cells using automated cell enrichment and concentration processes enables scalable, robust and cost-effective solutions

Kok, Dekkers et al.

Supplementary Figures

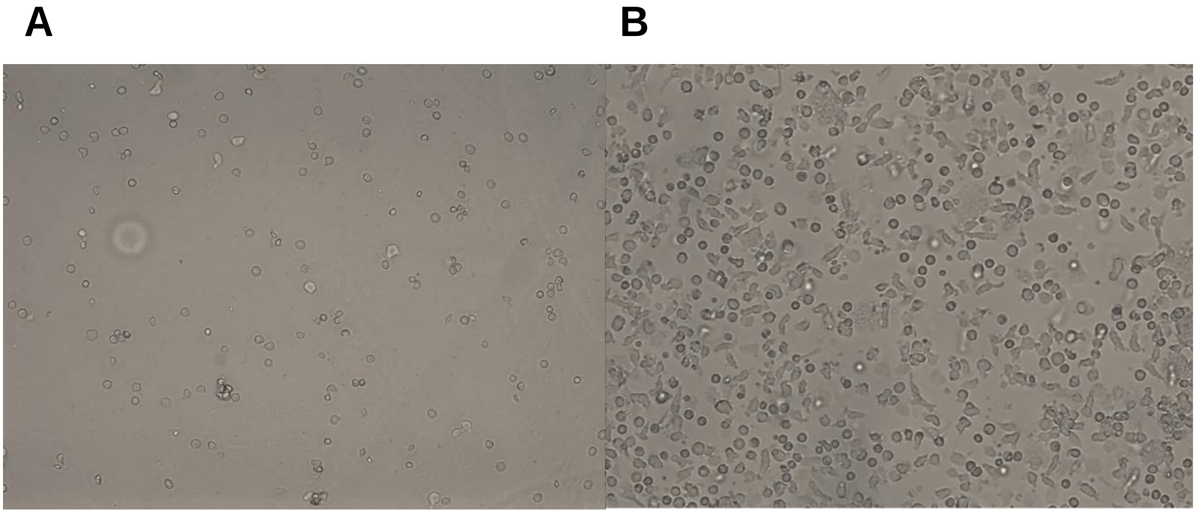

Figure S1 – Cell morphology. (**A**) Cell suspension containing CD34$^+$ cells 1 day after enrichment (20x magnification) and (**B**) cell suspension containing CD56$^+$ cells on harvest day before cell concentration (10x magnification).

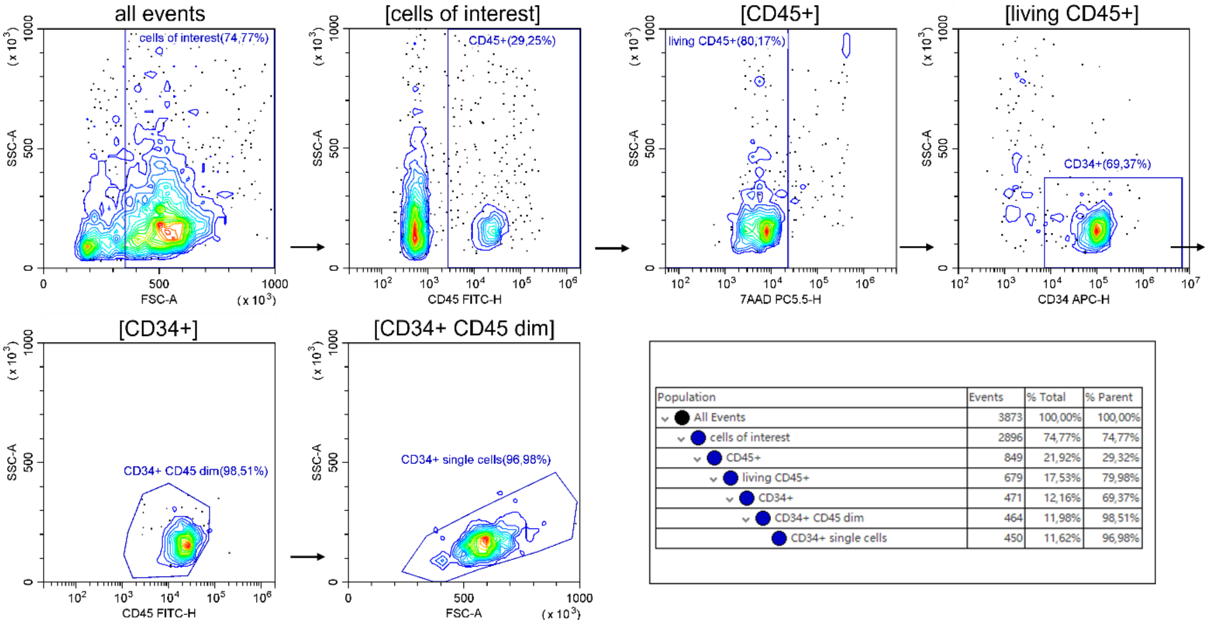

Population	Events	% Total	% Parent
All Events	3873	100,00%	100,00%
cells of interest	2896	74,77%	74,77%
CD45+	849	21,92%	29,32%
living CD45+	679	17,53%	79,98%
CD34+	471	12,16%	69,37%
CD34+ CD45 dim	464	11,98%	98,51%
CD34+ single cells	450	11,62%	96,98%

Figure S2 – Gating strategy for CD34$^+$ hematopoietic stem cell enumeration after enrichment from umbilical cord blood using the CliniMACS Prodigy®. Cells of interest are gated from FSC-A vs SSC-A, then CD45$^+$ cells are identified via CD45 FITC. Living 7-AAD PC5.5-negative cells (7AAD$^-$/CD45$^+$) are then gated on CD34 APC to detect CD34$^+$ cells and are further distinguished as CD34$^+$ CD45^{dim} cells on CD45 FITC with low SSC. Finally, CD34$^+$ single cells are detected from FSC-A vs SSC-A. FSC: forward scatter; SSC: side scatter; FITC: fluorescein isothiocyanate; 7-AAD: 7-amino-actinomycin D; PC5.5: phycoerythrin-cyanine 5.5; APC: allophycocyanin.

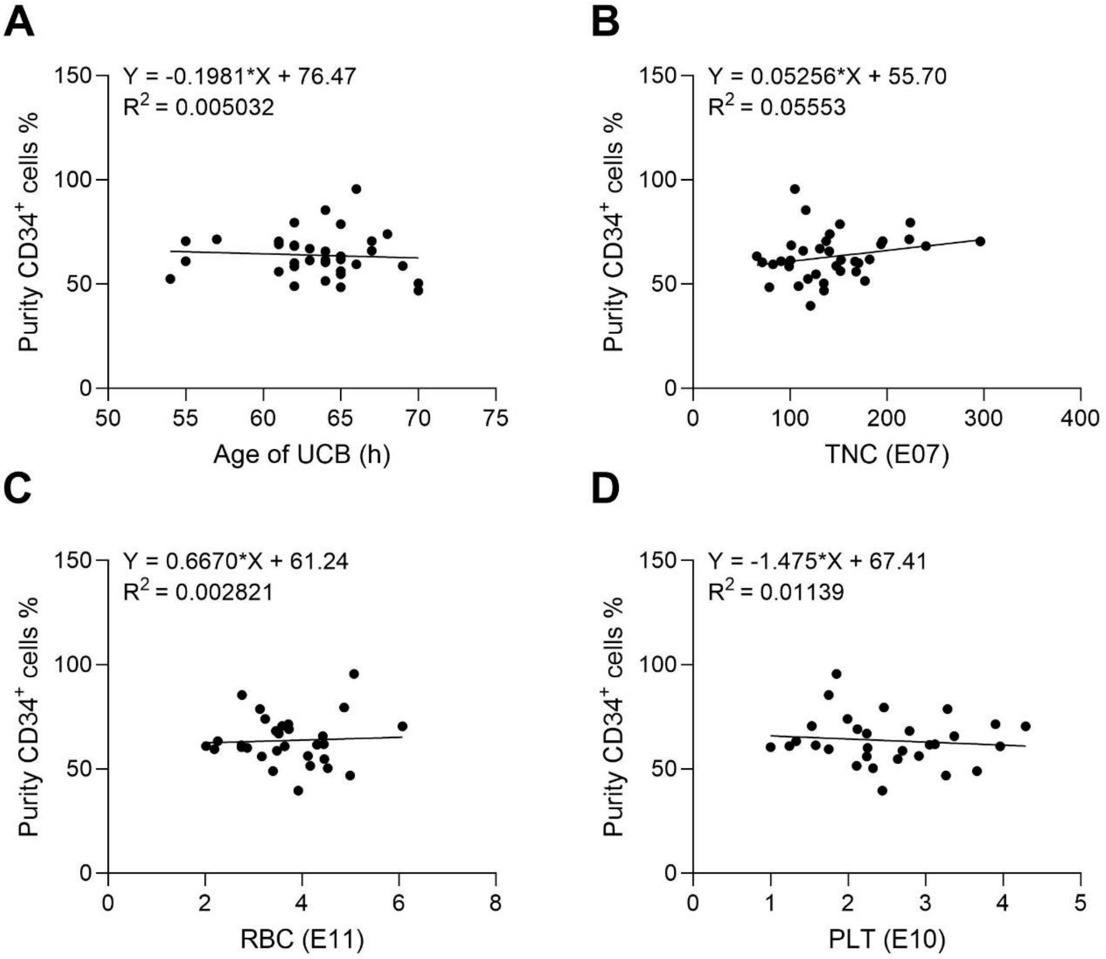

Figure S3 – $CD34^+$ hematopoietic stem cell purity after $CD34^+$ cell enrichment process using the CliniMACS Prodigy®. In all plots, $CD34^+$ cells were identified as living $CD34^+CD45^{dim}$ cells. Linear regression analysis between (**A**) age of UCB (n=35), (**B**) TNC (n=36) and (**C**) RBC (n=30) and (**D**) PLT (n=30) total content in UCB as reported by supplier and purity of $CD34^+$ hematopoietic stem cells after cell enrichment. All R^2 values show no correlation. UCB: umbilical cord blood; TNC: total nucleated cells; RBC: red blood cells; PLT: platelets.

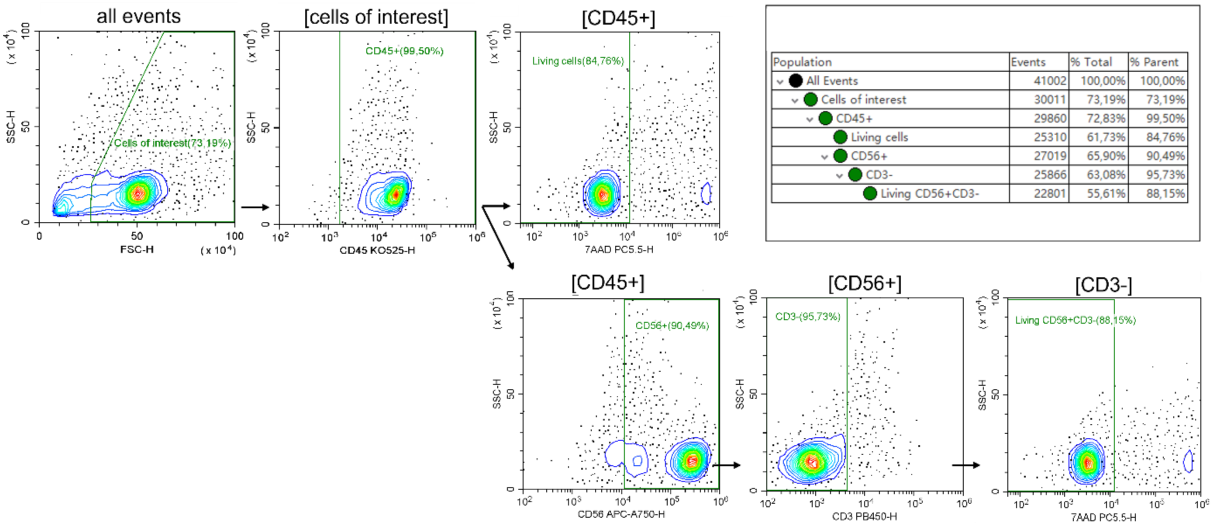

Population	Events	% Total	% Parent
All Events	41002	100,00%	100,00%
Cells of interest	30011	73,19%	73,19%
CD45+	29860	72,83%	99,50%
Living cells	25310	61,73%	84,76%
CD56+	27019	65,90%	90,49%
CD3-	25866	63,08%	95,73%
Living CD56+CD3-	22801	55,61%	88,15%

Figure S4 – Gating strategy for total and NK cell count before and after concentration from cell culture using the CliniMACS Prodigy®. Cells of interest are gated from FSC-H vs SSC-H, then total cells are identified as $CD45^{+}$ by CD45 KO525 staining and as living as 7-AAD PC5.5-negative ($7AAD^{-}$ $CD45^{+}$). NK cells are gated from $CD45^{+}$ cells, identified as $CD56^{+}$ cells by CD56 APC-A750 and as $CD3^{-}$ from CD3 PB-450. Living NK cells are then gated as $7AAD^{-}CD45^{+}CD56^{+}CD3^{-}$ cells. FSC: forward scatter; SSC: side scatter; KO: krome orange; 7-AAD: 7-amino-actinomycin D; PC5.5: phycoerythrin-cyanine 5.5; APC: allophycocyanin; PB: Pacific blue.

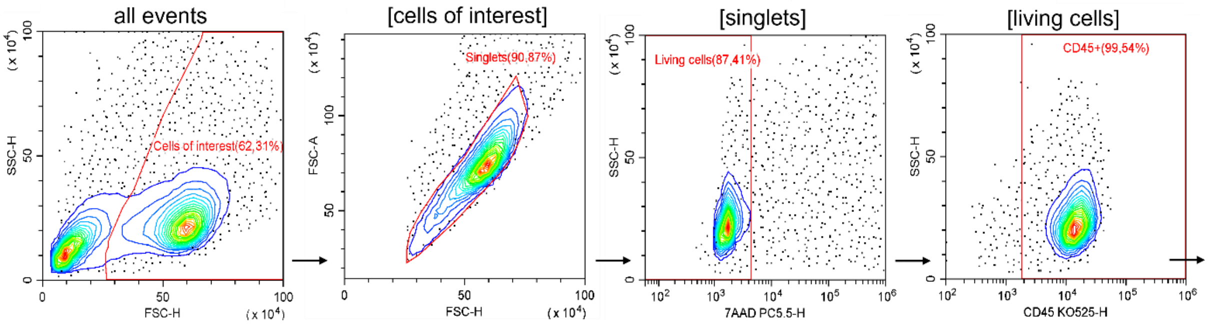

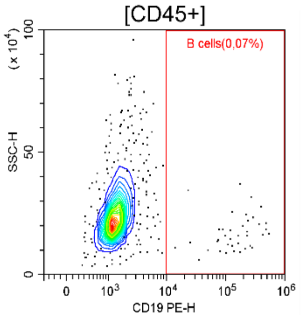

Population	Events	% Total	% Parent
All Events	198434	100,00%	100,00%
Cells of interest	123649	62,31%	62,31%
Singlets	112355	56,62%	90,87%
Living	98207	49,49%	87,41%
CD45+	97751	49,26%	99,54%
B cell	65	0,03%	0,07%

Figure S5 – Gating strategy for impurity analysis before and after concentration from cell culture using the CliniMACS Prodigy®. Cells of interest are gated from FSC-H vs SSC-H, then single cells are identified. Living cells are gated as 7AAD PC5.5-negative**.** Living CD45^{+} cells are selected from CD45 KO525 staining (7AAD^{-}CD45^{+}), then B cells are gated as CD19 PE-positive (7AAD^{-}CD45^{+}CD19^{+}). FSC: forward scatter; SSC: side scatter; 7-AAD: 7-amino-actinomycin D; PC5.5: phycoerythrin-cyanine 5.5; KO: krome orange; PE: phycoerythrin.
